# Aktuelle Trends der Inzidenz und Mortalität kutaner Lymphome in Deutschland

**DOI:** 10.1111/ddg.15904_g

**Published:** 2026-03-09

**Authors:** Khodr Cheikh El Najjarine, Hiltraud Kajüter, Ina Wellmann, Andreas Stang, Chalid Assaf

**Affiliations:** ^1^ Klinik für Dermatologie HELIOS Klinikum Krefeld Akademisches Lehrkrankenhaus der Universität Aachen, Krefeld; ^2^ Krebsregister Nordrhein‐Westfalen, Bochum; ^3^ Institut für Medizinische Informatik Biometrie und Epidemiologie Universitätsklinikum Essen Universität Duisburg‐Essen, Essen; ^4^ Institut für Molekulare Medizin Medical School Hamburg

**Keywords:** CBCL, CTCL, Epidemiologie, Inzidenz, kutane Lymphome, Mortalität, Register, Überleben, Cutaneous lymphomas, CTCL, CBCL, epidemiology, incidence, mortality, registry, survival

## Abstract

**Hintergrund:**

Primäre kutane Lymphome (*primary cutaneous lymphomas*, PCL) bestehen hauptsächlich aus kutanen T‐Zell‐Lymphomen (*cutaneous T‐cell lymphomas*, CTCL), gefolgt von kutanen B‐Zell‐Lymphomen (*cutaneous B‐cell lymphomas*, CBCL). Ziel dieser Studie war es, die Inzidenz und Überlebensraten von PCL in Deutschland zu ermitteln.

**Methoden:**

Wir analysierten Daten des nordrhein‐westfälischen Krebsregisters (2008–2021), das eine Bevölkerung von 18 Millionen Menschen umfasst. Es wurden altersstandardisierte Inzidenzraten und relative Überlebensraten berechnet.

**Ergebnisse:**

Die Analyse umfasste 3853 Patienten mit neu diagnostizierten PCL. Bei 69,5% dieser Patienten handelte es sich um CTCL und bei 24,9% um CBCL. Die altersstandardisierte Inzidenz von PCL betrug 10,8 pro Million Personenjahre. Die Inzidenz sowohl von CTCL als auch von CBCL nahm im Laufe der Zeit zu. PCL‐Fälle wurden auch bei Kindern festgestellt. Die relative Fünf‐Jahres‐Gesamtüberlebensrate für PCL betrug 89% (95% CI: 87–92%), wobei die Überlebensrate bei CTCL‐Patienten 91% (95% CI: 88–94%) und bei CBCL‐Patienten 86% (95% CI: 81–91%) betrug. Bei den Patienten mit Sézary‐Syndrom lag die relative Fünfjahresüberlebensrate bei 53% (95%: CI:29–77%).

**Schlussfolgerungen:**

Diese Studie ist bisher die größte bevölkerungsbasierte Analyse der PCL in Deutschland, die sowohl Erwachsene als auch Kinder umfasst. Die Inzidenz der PCL war höher als bisher berichtet. Darüber hinaus präsentieren wir die ersten Überlebensdaten für PCL in Deutschland, die unter anderem eine deutlich höhere Überlebenswahrscheinlichkeit für Patienten mit Sézary‐Syndrom zeigen.

## EINLEITUNG

Primäre kutane Lymphome (*primary cutaneous lymphomas*, PCL) sind eine heterogene Gruppe vonmalignen Hauterkrankungen, die sich hinsichtlich ihrer Symptome und Prognose stark unterscheiden.^1^, ^2^ Sie sind nach gastrointestinalen Lymphomen die zweithäufigste Form des extranodalen Non‐Hodgkin‐Lymphoms mit primärer Hautbeteiligung.^1^, ^3^, ^4^ PCLs sind durch das Vorliegen von ausschließlich hautbezogenen Symptomen zum Zeitpunkt der Diagnose definiert.

Die weltweite Inzidenz von PCL wird auf etwa 1 pro 100 000 Personen pro Jahr geschätzt. Davon sind 73% kutane T‐Zell‐Lymphome (*cutaneous T‐cell lymphomas*, CTCL), 22% kutane B‐Zell‐Lymphome (*cutaneous B‐cell lymphomas*, CBCL) und der Rest sind seltene Subtypen, die von natürlichen Killerzellen oder plasmazytoiden dendritischen Zellen ausgehen.^1^, ^2^, ^3^, ^4^ Die Subtypen der PCL haben unterschiedliche Prognosen, die von indolenten, langfristigen Verläufen bis zu aggressiven Erkrankungen mit schlechten Überlebenschancen reichen.^1^, ^5^, ^6^, ^7^

Epidemiologische Daten zu PCL aus bevölkerungsbasierten Studien waren aufgrund der Seltenheit und Heterogenität der Erkrankung bislang begrenzt. Darüber hinaus hat sich die Klassifizierung von PCL in den letzten 50 Jahren weiterentwickelt, was Vergleiche zwischen historischen und aktuellen Daten erschwert. Seit der Einführung des WHO‐EORTC‐Klassifizierungssystems im Jahr 2005, das klinische, histologische und molekulargenetische Merkmale berücksichtigt, ist die PCL‐Klassifizierung jedoch stabiler und weltweit vergleichbarer geworden.^1^

Jüngste Studien haben regionale Unterschiede in der Inzidenz und Subtypverteilung von PCL aufgezeigt.^6^ So sind beispielsweise seltene Subtypen wie EBV‐assoziierte NK/T‐Zell‐Lymphome in asiatischen Ländern häufiger anzutreffen, während andere Subtypen weltweit gleichmäßiger verteilt sind. Dobos et al. berichteten über eine erhöhte Inzidenz von PCL im französischen Hautlymphom‐Register zwischen 2005 und 2019.^8^ In den Niederlanden beobachteten Ottenvanger et al. zwischen 2000 und 2020 einen signifikanten Anstieg der Fälle von Mycosis fungoides und Sézary‐Syndrom.^7^

In Griechenland analysierten Kaliampou et al. die PCL‐Inzidenz und Subtypen im wichtigsten hämatopathologischen Zentrum Attikas von 2009 bis 2021.^8^ Ihre Ergebnisse deuten auf eine höhere nationale Inzidenz von 2,2 neuen Fällen pro 100 000 Personen (europäische Standardbevölkerung) hin – möglicherweise aufgrund verbesserter Diagnosemöglichkeiten. In ähnlicher Weise führten Titou et al. eine retrospektive Analyse von 114 Fällen von Mycosis fungoides (1993–2022) durch und stellten eine höhere Prävalenz bei älteren Männern fest, mit einem Gesamtüberleben von 85,7% nach 5 Jahren, 74,6% nach 10 Jahren und 61,4% nach 20 Jahren.^9^

Es wurden auch regionale Unterschiede in der Altersverteilung festgestellt. Eine Studie aus Saudi‐Arabien berichtete über ein Durchschnittsalter von 41 Jahren bei CTCL‐Patienten, verbunden mit einer bemerkenswert hohen pädiatrischen Inzidenz von 12,8%.^10^ Diese Rate ist im Fernen Osten sogar noch höher, wo bis zu 25% der CTCL‐Fälle bei Kindern auftreten.^11^

Die bisher aktuellsten groß angelegten registerbasierten epidemiologischen Daten zu PCL in Deutschland wurden 2007 von Assaf et al. veröffentlicht und basieren auf einem Register mit 998 Patienten aus 26 dermatologischen Abteilungen.^12^ Diese Studie lieferte grundlegende Daten zur Demografie der Patienten und zur Verteilung der Subtypen.

In der vorliegenden Studie zeigen wir erstmals in Deutschland eine umfassende Analyse der PCL‐Inzidenz und der relativen Überlebensrate – einschließlich subtypspezifischer Ergebnisse. Die Daten stammen aus dem Krebsregister Nordrhein‐Westfalen, das eine Bevölkerung von 18 Millionen Menschen erfasst und gemeldete Fälle von Hautlymphomen aus den Jahren 2008 bis 2021 umfasst.

## MATERIAL UND METHODEN

Das Krebsregister Nordrhein‐Westfalen (LKR NRW) erfasst eine Bevölkerung von etwa 18 Millionen Menschen, was etwa 22% der Gesamtbevölkerung Deutschlands entspricht. Die Meldung von Krebserkrankungen ist für alle diagnostizierenden und behandelnden Ärzte, einschließlich Pathologen und Dermatopathologen, obligatorisch. Die Vollständigkeit des Registers wird regelmäßig vom Zentrum für Krebsregisterdaten am Robert Koch‐Institut überprüft und liegt für das LKR NRW bei über 95%.

Fälle von PCL wurden anhand der ICD‐O‐Morphologiecodes 9590/3–9729/3 (Hodgkin‐ und Non‐Hodgkin‐Lymphome) und der ICD‐O‐Topografie‐Codes C44 (Haut), C51 (Vulva), C60 (Penis) oder C63.2 (Hodensack) identifiziert, basierend auf der Internationalen Klassifikation der Krankheiten für Onkologie, 3. Auflage (ICD‐O‐3).

Die Analyse wurde weiter auf Fälle beschränkt, die gemäß der 10. Revision der Internationalen Klassifikation der Krankheiten (ICD‐10) mit C82–C88 kodiert waren. Der Datensatz umfasst PCL‐Fälle, die zwischen Januar 2008 und Dezember 2021 diagnostiziert wurden.

Wir analysierten PCL insgesamt, sowie CTCL und CBCL separat. Diese Gruppen wurden nach ICD‐10‐Codes weiter unterteilt. Zu den CTCL zählen Mycosis fungoides (MF, C84.0), das Sézary‐Syndrom (SS, C84.1) sowie primäre kutane CD30‐positive T‐Zell‐Proliferationen (pcCD30^+^LPD, C86.6). Die CBCL umfassen das primäre kutane follikuläre Lymphom (pcFL, C82), das primäre kutane diffuse großzellige B‐Zell‐Lymphom (pcDLBCL, C83.3) und das primäre kutane Marginalzonenlymphom (pcMZL, C88.4).

Die folgenden Variablen wurden in der Analyse berücksichtigt: Geschlecht, Alter bei der Diagnose, Histopathologie, Entität, anatomische Lage, Todesdatum und die diagnostische Klassifizierung gemäß den WHO‐EORTC‐Kriterien von 2018.^1^

### Statistische Methoden

Die altersstandardisierten Inzidenzraten für den Untersuchungszeitraum Januar 2008 bis Dezember 2021 wurden unter Verwendung der „alten“ europäischen Standardbevölkerung berechnet.^13^ Fälle, bei denen die Sterbeurkunde die einzige Informationsquelle war (DCO), wurden in die Inzidenzberechnungen einbezogen, jedoch aufgrund fehlender detaillierter klinischer Daten aus den Überlebensanalysen ausgeschlossen.

Wir schätzten die relative Fünfjahresüberlebensrate unter Verwendung des Periodenanalyseansatzes auf der Grundlage der Überlebensdaten für den Kalenderzeitraum 2017–2021.^14^, ^15^ Diese Methode berücksichtigt nicht nur Patienten, die zwischen 2017 und 2021 diagnostiziert wurden, sondern auch diejenigen, die in den vorangegangenen 5 Jahren diagnostiziert wurden und bis in den Zeitraum 2017–2021 überlebten, wodurch aktuellere Überlebensschätzungen erzielt werden.

Um die Genauigkeit unserer Schätzungen zu beurteilen, haben wir Standardfehler (SE) und Konfidenzintervalle (KI) berechnet und angegeben. Da das primäre Ziel dieser Studie die Schätzung und nicht die Hypothesenprüfung ist, vermeiden wir es, die statistische Signifikanz hervorzuheben, und konzentrieren uns stattdessen auf die Genauigkeit und Validität unserer Ergebnisse, um das Risiko einer Veröffentlichungsverzerrung zu verringern.^16^, ^17^

Alle Analysen wurden mit der SAS‐Software Version 9.4 (SAS Institute, Cary, NC) durchgeführt.

## ERGEBNISSE

### Inzidenz und Demografie

Von 2008 bis 2021 wurden insgesamt 3853 Fälle von PCL von Klinikern, Pathologen und Dermatopathologen an das Krebsregister Nordrhein‐Westfalen gemeldet. Davon waren 2680 Fälle (69,6%) CTCL, 960 Fälle (24,9%) CBCL und 213 Fälle (5,5%) nicht näher bezeichnete oder seltene Subtypen (Tabelle 1).

**TABELLE 1:** Anzahl und altersstandardisierte Inzidenzraten der erfassten kutanen Lymphomfälle im bevölkerungsbezogenen Krebsregister Nordrhein‐Westfalen, Deutschland, 2008–2021.

	Gesamt				Männer				Frauen			
Entität der kutanen Lymphome	N	%	ASR	SE	N	%	ASR	SE	N	%	ASR	SE
Gesamt	3853		10,8	0,18	2386		14,2	0,30	1467		7,7	0,22
**Kutane T‐Zell‐Lymphome**	2680	100	7,5	0,15	1756	100	10,4	0,26	924	100	5,1	0,18
Mycosis fungoides (C84.0)	1827	68,2	5,1	0,13	1246	71,0	7,4	0,22	581	62,9	3,2	0,14
Sézary syndrome (C84.1)	94	3,5	0,2	0,02	45	2,6	0,2	0,03	49	5,3	0,2	0,03
Primär kutane CD30‐positive T‐Zell‐Proliferationen. (C86.6)	91	3,4	0,3	0,03	49	2,8	0,3	0,04	42	4,5	0,3	0,04
Andere T‐Zell‐Lymphome	668	24,9	1,9	0,08	416	23,7	2,5	0,13	252	27,3	1,4	0,10
**Kutane B‐Zell‐Lymphome**	960	100	2,7	0,09	516	100	3,2	0,15	444	100	2,2	0,11
Follikuläres Lymphom (C82)	370	38,5	1,1	0,06	209	40,5	1,3	0,10	161	36,3	0,9	0,07
Diffus großzelliges B‐Zell Lymphom (C83.3)	254	26,5	0,6	0,04	125	24,2	0,7	0,06	129	29,1	0,4	0,04
Marginalzonen Lymphom (C88.4)	320	33,3	1,0	0,06	171	33,1	1,1	0,09	149	33,6	0,8	0,07
Andere B‐Zell‐Lymphome	16	1,7	0,0	0,01	11	2,1	0,1	0,02	5	1,1	0,0	0,01
**Andere & unspezifizierte Lymphome**	213		0,6	0,04	114		0,7	0,06	99		0,5	0,05
**Alter bei Diagnose: Median (IQR)**	68,8 (57,4; 78,4)					68,7 (57,3; 77,9)				69,0 (57,4; 79,3)		

Alle Raten sind als Fälle pro Million Personenjahre ausgedrückt; die Raten sind auf die alte europäische Standardbevölkerung normiert. Abkürzungen: ASR ‐ altersstandardisierte Inzidenzrate, SE ‐ Standard Error (Standardfehler), IQR ‐ interquantile range (Interquantilbereich)

**ABBILDUNG 1:**
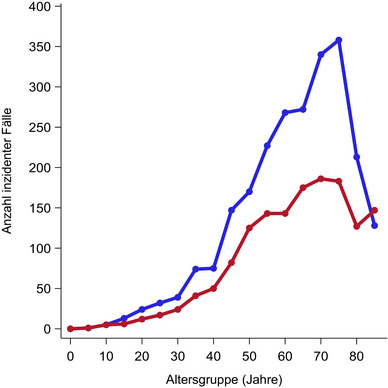
Altersverteilung männlicher und weiblicher Inzidenzfälle kutaner Lymphome in Nordrhein‐Westfalen, Deutschland, 2008–2021. Männer, blaue Kurve; Frauen, rote Kurve. Das Medianalter bei Diagnose sowie das 25. und 75. Perzentil betrug bei Männern 67,3 Jahre (55,3; 76,3) und bei Frauen 67,6 Jahre (55,1; 77,3).

Die altersstandardisierte Inzidenzrate von PCL betrug 14,2 pro Million Personenjahre bei Männern und 7,7 bei Frauen, mit einer Gesamtinzidenz von durchschnittlich 10,8 pro Million Personenjahre. Die Inzidenzraten stiegen exponentiell mit dem Alter an und erreichten ihren Höhepunkt bei Personen im Alter von 60 Jahren und älter. Das mediane Alter bei der Diagnose betrug 66,9 Jahre (67,6 Jahre bei Männern, 65,7 Jahre bei Frauen). Bemerkenswert ist, dass 0,8% der Fälle bei Personen unter 20 Jahren auftraten (Abbildung 1).

Bei Männern divergierten die altersspezifischen Inzidenzraten für CTCL und CBCL mit zunehmendem Alter erheblich und erreichten ihren Höhepunkt insbesondere in der Altersgruppe der 70‐ bis 74‐Jährigen. Im Gegensatz dazu wiesen jüngere Frauen (im Alter von 25 bis 49 Jahren) höhere Inzidenzraten für CTCL im Vergleich zu CBCL auf, obwohl sich dieser Unterschied im Alter von 55 bis 59 Jahren verringerte. Bei CTCL vergrößerte sich der Geschlechtsunterschied in der Inzidenz mit zunehmendem Alter, ein Muster, das bei CBCL nicht zu beobachten war (Abbildungen S1, S2 im Online‐Supplement).

Insgesamt wurden 2386 Männer (61,9%) und 1467 Frauen (38,1%) mit PCL diagnostiziert, was einem Verhältnis von Männern zu Frauen von 1,6:1 entspricht. Die meisten PCL‐Subtypen traten häufiger bei Männern auf. Unter den CTCL war die Mycosis fungoides der häufigste Subtyp (Tabelle 1). Bei CBCL waren die indolenten Subtypen – pcFL und pcMZL – am häufigsten, gefolgt vom aggressiven pcDLBCL (Tabelle 1).

### Zeitliche Trends in der Inzidenz

**ABBILDUNG 2:**
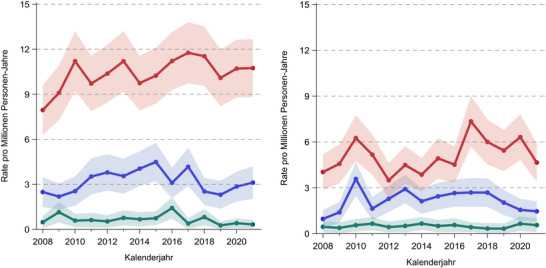
Altersstandardisierte Inzidenzraten kutaner Lymphome in Nordrhein‐Westfalen, Deutschland, 2008–2021, nach Geschlecht. Rote Kurven, kutane T‐Zell‐Lymphome (CTCL); blaue Kurven, kutane B‐Zell‐Lymphome (CBCL); grüne Kurven, andere kutane Lymphome.

Von 2008 bis 2021 stiegen die altersstandardisierten Inzidenzraten sowohl für CTCL als auch für CBCL bei Männern und Frauen an (CTCL – Männer: von 7,95 auf 10,74 pro 100 000 Personenjahre [+35,1%], Frauen: von 4,03 auf 4,65 pro 100 000 Personenjahre [+15,5%]; CBCL‐Männer: von 2,49 auf 3,11 pro 100 000 Personenjahre [+25,0%], Frauen: von 0,96 auf 1,46 pro 100 000 Personenjahre [+5,3%]). In Bezug auf die absoluten Fallzahlen stieg die Zahl der CTCL‐Fälle von 93 auf 123 (Männer) und von 54 auf 65 (Frauen), und die Zahl der CBCL‐Fälle stieg von 25 auf 35 Fälle (Männer) und von 12 auf 26 Fälle (Frauen) pro Jahr (Abbildung 2).

### Überlebensanalyse

Die relative Fünfjahresüberlebensrate wurde für den Zeitraum 2017–2021 geschätzt (Tabelle 2). Die relative Fünfjahresüberlebensrate für PCL betrug insgesamt 89,0%, wobei die Überlebensrate bei Männern (90,8%) höher war als bei Frauen (86,1%).

**TABELLE 2:** Fünf‐Jahres‐relative Überlebensschätzungen (%) für 2017–2021 bei Patienten mit neu diagnostizierten kutanen Lymphomen in Nordrhein‐Westfalen, Deutschland.

	Gesamt		
Kutane Lymphome – Subtypen	n	Schätzung	95%‐Kl
Insgesamt	2674	89,0	(86,5; 91,5)
Insgesamt, Männer	1656	90,8	(87,6; 94)
Insgesamt, Frauen	991	86,1	(82,1; 90,1)
***Kutane T‐Zell‐Lymphome***	1835	91,0	(88,1; 93,9)
Mycosis fungoides (C84.0)	1266	96,2	(93; 99,4)
Sézary‐Syndrom (C84.1)	42	53,0	(28,9; 77,2)
Primäre kutane CD30‐positive T‐Zell‐Proliferationen (C86.6)	66	98,2	(85,6; 110,7)
Andere T‐Zell‐Lymphome	461	80,2	(74; 86,3)
***Kutane B‐Zell‐Lymphome***	*677*	*86,0*	*(80,7; 91,2)*
Follikuläre Lymphome (C82)	288	100,6	(95,2; 106)
Diffus großzellige B‐Zell‐Lymphome (C83.3)	146	55,5	(42,3; 68,7)
Marginalzonen‐Lymphome (C88.4)	233	92,3	(84,1; 100,4)
Andere B‐Zell‐Lymphome	10	–	–
**Andere und nichtspezifizierte Lymphome**	135	80,0	(68; 92)

*Abk*.: KI, Konfidenzintervall

**ABBILDUNG 3:**
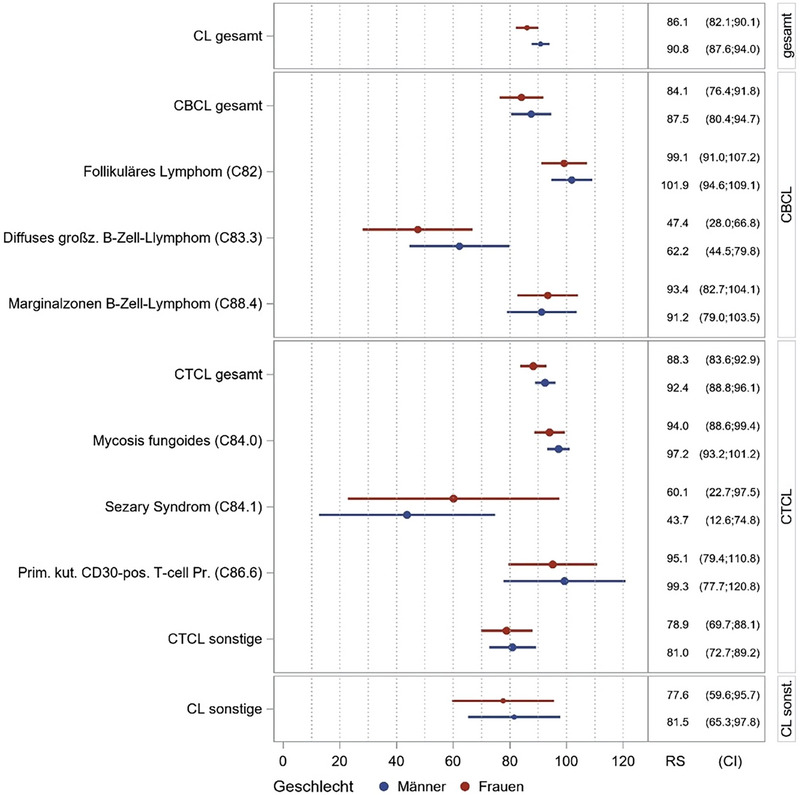
Relatives Überleben, 2017–2021. CL, kutanes Lymphom; CTCL, kutanes T‐Zell‐Lymphom; CBCL, kutanes B‐Zell‐Lymphom; C82, follikuläres Lymphom; C83.3, diffus großzelliges B‐Zell‐Lymphom; C88.4, Marginalzonenlymphom; C84.0, Mycosis fungoides; C84.1, Sézary‐Syndrom; C86.6, primär kutane CD30‐positive T‐Zell‐Proliferationen.

Die Überlebensraten nach PCL‐Subtypen zeigten für CTCL insgesamt 91,0 % (MF 96,2 %, SS 53,0 %, pcCD30^+^LPD 98,2 %, nicht spezifiziertes CTCL 90,2 %). Für CBCL betrug die Überlebensrate insgesamt 86,0 % (pcFL 100 %, pcDLBCL 55,5 %, pcMZL 92,3 %). Aufgrund der begrenzten Zahl nicht näher bezeichneter CBCL‐Fälle konnte für diese Gruppe keine verlässliche Fünfjahresüberlebensrate geschätzt werden (Tabelle 2, Abbildung 3).

## DISKUSSION

Wir präsentieren bevölkerungsbasierte Inzidenz‐ und Überlebensdaten für PCL von 2008 bis 2021, die aus dem LKR NRW stammen – dem größten Krebsregister Deutschlands, das etwa 18 Millionen Einwohner umfasst. Dieser Datensatz ermöglicht eine eingehende Analyse der epidemiologischen Muster und zeitlichen Veränderungen sowohl bei CTCL als auch bei CBCL.

Die altersstandardisierte Inzidenzrate für PCL in Nordrhein‐Westfalen lag bei 1,08 pro 100 000 Personenjahre und war damit höher als in Frankreich (0,96) und den USA (0,87) und entsprach anderen europäischen Registern, lag jedoch immer noch unter der Rate in Griechenland (2,2).^6^, ^8^, ^18^ Dies unterstreicht die regionalen Unterschiede in der Inzidenz, die möglicherweise auf Unterschiede in der Diagnostik, der Vollständigkeit der Register oder den zugrunde liegenden Risikofaktoren der Bevölkerung zurückzuführen sind.

In unserer Kohorte machte CTCL 69,9% aller PCL‐Fälle aus, was mit früheren Ergebnissen (70–85%) übereinstimmt.^6^, ^12^, ^19^ Die CTCL‐Inzidenzrate in unseren Daten (7,5 pro Million Personenjahre) war deutlich höher als die im SEER‐Programm gemeldeten Raten von 1,4 pro Million^20^, ^21^, ^22^, ^23^, ^24^ (*Surveillance, Epidemiology, and End Results*, ein bevölkerungsbasiertes Krebsregisterprogramm, das vom National Cancer Institute in den Vereinigten Staaten geführt wird) und anderen europäischen Datensätzen gemeldeten Raten deutlich höher.^6^, ^12^ Die Dominanz von MF unter den CTCL‐Subtypen (68,2%) entspricht den europäischen Trends,^8^, ^10^, ^15^ übersteigt jedoch den vom US‐amerikanischen SEER‐Programm gemeldeten Anteil von 56,6%.^24^

Diese Diskrepanz könnte auf Unterschiede in der Klassifizierungsgenauigkeit zurückzuführen sein. Die SEER‐Daten zeigen einen größeren Anteil von CTCL‐Fällen, die als „Sonstige“ kategorisiert sind, möglicherweise aufgrund unvollständiger diagnostischer Informationen. Im Gegensatz dazu könnte unser Register, das sich stark auf pathologische Befunde stützt, histologisch bestätigte Entitäten wie das periphere T‐Zell‐Lymphom (PTCL) überrepräsentieren, während Diagnosen wie CD30^+^LPD, die auf dem klinischen Kontext beruhen, unterrepräsentiert sind.

Darüber hinaus haben SEER‐Daten geografische Unterschiede in der MF‐Inzidenz gezeigt – mit einer mehr als doppelt so hohen Rate in Ballungsräumen im Vergleich zu nichtstädtischen Gebieten –, was auf eine mögliche Rolle von Umweltrisikofaktoren in der Pathogenese der MF hinweist.^24^

CBCL machten in unserer Studie 24,9% der PCL aus, was nur geringfügig über den in der bisherigen Literatur angegebenen 24% lag.^6^, ^12^ Die häufigsten CBCL‐Subtypen waren pcMZL, pcFL und pcDLBCL.

Diese Studie ist eine der ersten in Deutschland, die relative Fünfjahresüberlebensraten für PCL‐Subtypen in einer bevölkerungsbasierten Umgebung liefert. Die relative Gesamtüberlebensrate für PCL betrug 89,0%, wobei es je nach Subtyp erhebliche Unterschiede gab: CTCL: 91,0%; MF: 96,2%; CD30^+^LPD: 98,2%; SS: 53,0%.

Unsere Überlebensraten für MF und CD30^+^LPD stimmen mit den Ergebnissen der niederländischen und österreichischen Hautlymphom‐Gruppen (95% und 88%),^1^, ^25^, ^26^ und der marokkanischen Studie (85,1%),^9^ überein.

Interessanterweise ist unsere erfasste Überlebensrate von 53,0% bei SS deutlich höher als historische Schätzungen (20–42%) und entspricht weitgehend einer aktuellen internationalen multizentrischen Studie (53,4%), was auf verbesserte Ergebnisse in den letzten Jahren hindeutet. Diese Verbesserung könnte auf die Einführung neuartiger Therapien wie Brentuximab Vedotin (Anti‐CD30) und Mogamulizumab (Anti‐CCR4), die beide seit 2018 zugelassen sind, sowie auf den zunehmenden Einsatz von Kombinationstherapien, zum Beispiel extrakorporale Photopherese mit Retinoiden oder Interferon, zurückzuführen sein.^27^, ^28^, ^29^

Für CBCL betrug die relative Fünfjahresüberlebensrate 86,0%, für pcMZL 92,3%, für pcFL 100% und für pcDLBCL 55,5%. Unsere Überlebensschätzungen für pcMZL und pcFL sind mit früheren europäischen Studien vergleichbar (99% und 95%).^1^, ^3^, ^9^ Die leicht verringerte relative Überlebensrate bei pcMZL könnte darauf zurückzuführen sein, dass Fälle mit sekundärer Hautbeteiligung infolge einer systemischen Erkrankung einbezogen wurden, wie bereits in früheren Arbeiten vermutet.^30^

Im Vergleich zu internationalen Daten zeigten pcDLBCL‐Patienten in unserem Register eine höhere Überlebensrate (55,5%) als diejenigen in der französischen Studiengruppe (41%), aber eine niedrigere als in den SEER‐Daten (64,7%). Die Gründe für diese Diskrepanz sind unklar, insbesondere da die Behandlungsprotokolle zwischen Europa und den USA bei dieser Entität weitgehend harmonisiert sind.^31^, ^32^, ^33^

Unsere Daten zeigen eine steigende Inzidenz von CTCL und CBCL bei beiden Geschlechtern über den 14‐jährigen Studienzeitraum hinweg – was den Trends in den US‐amerikanischen SEER‐Daten entspricht.^20^, ^21^, ^22^, ^23^, ^24^ Im Gegensatz dazu blieb die Inzidenz anderer seltener PCL‐Typen stabil. Diese Ergebnisse stimmen weitgehend mit unseren früheren Arbeiten auf der Grundlage von gesetzlichen Krankenversicherungsansprüchen überein,^34^, ^35^ obwohl es bemerkenswerte Unterschiede in der Subtypverteilung und der Trenderkennung gibt, die wahrscheinlich auf Einschränkungen hinsichtlich der Datenqualität und Granularität in der forschungsbasierten Forschung zurückzuführen sind.

Die Studie umfasst einschließlich die Jahre 2019–2021 und überschneidet sich damit mit der COVID‐19‐Pandemie. Frühere Untersuchungen haben einen Anstieg der MF/SS‐Diagnosen im fortgeschrittenen Stadium während dieses Zeitraums festgestellt.^36^ Aufgrund der sehr geringen Inzidenz von Hautlymphomen und den daraus resultierenden großen jährlichen Schwankungen der Inzidenzraten ist es jedoch praktisch unmöglich zu bestimmen, ob die Pandemie oder die damit verbundenen Lockdowns zu einem Rückgang der Diagnosen oder der Versorgung geführt haben, wie dies bei häufigen Krebsarten wie Melanomen und Nicht‐Melanom‐Hautkrebs der Fall war.^37^, ^38^, ^39^, ^40^ Darüber hinaus fehlen in bevölkerungsbezogenen Krebsregistern – wie dem in dieser Studie verwendeten – häufig detaillierte klinische Informationen zu kutanen Lymphomen, insbesondere zur Stadieneinteilung. Daher können mögliche Verschiebungen in der Stadieneinteilung aufgrund der Pandemie nicht aussagekräftig bewertet werden.

Eine wesentliche Einschränkung dieser Studie ist das Fehlen detaillierter klinischer Daten (zum Beispiel Stadieneinteilung, Behandlungsschemata, Fortschreiten der Erkrankung), das derzeit durch die laufende Einführung einer bevölkerungsbezogenen klinischen Krebsregistrierung behoben wird. Die Verknüpfung von klinischen Daten mit Registerdaten wird die Vollständigkeit und Qualität der Daten verbessern und differenziertere Analysen ermöglichen.

Zusammenfassend zeigt unsere Studie *(1)* eine steigende Inzidenz von PCL in Deutschland mit detaillierten Einblicken in altersspezifische Muster, einschließlich seltener pädiatrischer Fälle, *(2)* einen Anstieg der subtypspezifischen Diagnosen, insbesondere innerhalb des CBCL, was auf eine verbesserte diagnostische Genauigkeit im Zeitverlauf hinweist, und *(3)* erstmals in Deutschland bevölkerungsbasierte Überlebensdaten für PCL, einschließlich verbesserter Ergebnisse beim Sézary‐Syndrom, wahrscheinlich infolge therapeutischer Fortschritte.

Diese Ergebnisse liefern wertvolle epidemiologische Erkenntnisse zu PCL und unterstützen die Weiterentwicklung gezielter Strategien in der klinischen Praxis und der Krebsbekämpfung.

## DANKSAGUNG

Open access Veröffentlichung ermöglicht und organisiert durch Projekt DEAL.

## INTERESSENKONFLIKT

C.A. erhielt Beratungshonorare von 4SC, Helsinn, Innate Pharma, Kyowa Kirin, Recordati Rare Diseases und Takeda Pharmaceuticals. Alle anderen Autoren erklären, dass keine Interessenkonflikte bestehen

## Supporting information



Supplementary information

Supplementary information

Supplementary information
